# Storage Stability and Flavor Change of Marinated Pork

**DOI:** 10.3390/foods11131825

**Published:** 2022-06-21

**Authors:** Yin Zhang, Hui Li, Yingjie Zhang, Linguo Wang, Pengcheng Zhang, Jianlin Jia, Haichuan Peng, Qin Qian, Jiaming Zhang, Zhongli Pan, Dayu Liu, Liming Zhao

**Affiliations:** 1 Meat Processing Key Laboratory of Sichuan Province, Chengdu University, Chengdu 610106, China; lh13096297263@163.com (H.L.); fireboltzyj@126.com (Y.Z.); 15196956065@163.com (L.W.); zhangpc46@163.com (P.Z.); 19982048990@163.com (J.J.); phc960205@163.com (H.P.); 13541705752@163.com (Q.Q.); jasminejjjjjj@163.com (J.Z.); liudy1014@163.com (D.L.); 2 Department of Biological and Agricultural Engineering, University of California, Davis, One Shields Avenue, Davis, CA 95616, USA; zlpan@ucdavis.edu; 3 R&D Center of Separation and Extraction Technology in Fermentation Industry, State Key Laboratory of Bioreactor Engineering, East China University of Science and Technology, Shanghai 200237, China; zhaoliming@ecust.edu.cn

**Keywords:** marinated pork, chili essential oils, pepper essential oils, flavor, umami taste

## Abstract

To evaluate the storage stability and flavor changes of marinated pork treated with chili and pepper essential oils, the contents of total sulfhydryl, malondialdehyde, total volatile base nitrogen (TVBN), Ca^2+^ATPase activity, and total viable counts of marinated pork were determined. Further, the non-volatile (umami, numb, and spicy) and volatile flavor compounds of marinated pork were analyzed. Based on the results, the chili and pepper essential oils had limited effects on the storage stability of marinated pork. However, these essential oils could inhibit the oxidation of lipids and proteins and reduce the number of microorganisms and TVBN in marinated pork within 6 days. The non-volatile flavors of the marinated pork decreased as the refrigeration time increased. It was concluded that the decomposition of umami-enhancing nucleotides (GMP, IMP, XMP), the number of flavor substances (hydroxyl-α-sanshool, hydroxyl-β-sanshool), and spicy (capsaicin) tasting compounds caused the decrease in non-volatile flavors.

## 1. Introduction

Marinated meat products are semi-finished products or pre-finished products made with meat or its edible by-products as the main raw materials and processed by rolling or marinating with salt and other condiments [1]. Since the commercialization of marinated meat products in the 2010s, these products have attracted extensive attention from consumers and meat producers, and with the further development of online ordering and takeout options, the market share of marinated meat products is expected to expand further [2]. However, there are some technical problems in the production and storage of marinated meat products, such as unstable conditioning processes, decline of edible quality during storage, short storage time, and flavor attenuation [1,3]. In particular, flavor attenuation significantly decreases the market price of marinated meat products. To resolve these problems, researchers have attempted to improve the processing of marinated meat products. Fu, Liu, Gao, Yang, and Huangpu [4] revealed that sterilization temperature and time markedly influenced the quality of sauced beef, with the marinated beef sterilized at 115 °C for 30 min being superior to those subjected to other conditions in terms of color, chewiness, elasticity, and sensory quality. Li et al. [4] compared the impact of storage temperature (4 °C and −4 °C) on the quality of marinated yak. According to their results, as storage time increased, cooking loss of the marinated yak increased at 4 °C, chewiness of the marinated yak increased at −4 °C, and redness and hardness of the marinated yak decreased at 4 °C and −4 °C. Recently, Tong et al. [5] evaluated the effect of *litsea pungens* Hemsl. oil on the quality of cold-stored marinated yaks. *Litsea pungens* Hemsl. oil not only extended the shelf life of the marinated yak by 6 days, but also increased its water holding capacity, shear force, and redness. Tshabalala, Kabelinde, Foka, Ateba, and Manganyi [6] used clove extracts to inhibit meat-borne bacteria (*Escherichia coli*, *Enterococcus species* and *Staphylococcus aureus*). These clove extracts were found to display the highest inhibitory effect against *E. coli,* and the organoleptic characteristics of the marinated meat products were found to slightly decrease. The terpenoid compounds of capsaicin and carotenoids such as vitamins C, E, and provitamin A in powder or extract of chili and pepper make them show not only a flavoring effect, but also antibacterial and antioxidant effects [6,7,8]. Minh [9] added 1.5% chili powder to Vietnamese fermented pork roll (Nem Chua) and found that chili powder inhibited the growth of *enterobacteriaceae* and retarded the growth of protein oxidation. Soodsawaeng et al. [10] found that chili spur pepper and lemongrass extract (80 mg/mL) had a weak effect in lowering the total viable count in dried squid during a 28-day storage at room temperature. Wang et al. [11] added 0.3% and 0.5% pepper (*Zanthoxylum bungeanum Maxim.*) essential oil to rabbit meat patty and found the essential oil had inhibited the lipid and protein oxidation, microbial growth, and formation of TVB-N during the 12 days storage time. Although recent investigations have explored preparation processes and methods to extend the storage stability of marinated meat products, limited studies have been carried out to evaluate storage stability and changes in the flavor of marinated pork during cold storage. Marinated pork is the most widely consumed meat product [1,12]. To ensure marinated pork products possess a good taste, chili (*Capsicum annuum L.*), pepper (*Zanthoxylum bungeanum*), monosodium glutamate (MSG), and other condiments are added to these products in China [13,14,15,16,17]. As condiments mix with other ingredients and remain in an acidic environment owing to the metabolism of meat after slaughtering, the changes that occur owing to the added condiments have rarely been investigated. Flavor is very important for maintaining the edible quality of meat products [18], especially for marinated meat products that must be stored for a certain period before consumption. Therefore, the aim of this investigation was to evaluate the storage stability and flavor changes of marinated pork treated with chili and pepper essential oils. The findings of this study will provide a theoretical basis for improving current production processes and should be beneficial for the development of new marinated meat products.

## 2. Materials and Methods

### 2.1. Materials

Pork ham bicipital muscle was sampled from five male hybrid white pigs (Meishan pig × Chenghua pig × Landrace pig), aged one year old (weight 75 ± 5 kg) and with the same feeding conditions. Ham bicipital muscles were collected approximately within 24 h after slaughtering at Chengdu hope Food Co., Ltd. (Chengdu, China), refrigerated at 4 °C temperature and transported it to our laboratory. Chili (*Capsicum annuum* L.) essential oil was bought from Jiangxi Weiyuan Food Co., Ltd. (Weiyuan, China). Pepper (*Zanthoxylum bungeanum*) essential oil was purchased from Chongqing Huacui Biotechnology Co., Ltd. (Chongqing, China). Methanol, potassium hydroxide, potassium dihydrogen phosphate, trichloroacetic acid (TCA), thiobarbituric acid, hydrochloric acid, and other chemical reagents were purchased from Chengdu Cologne Chemicals Co., Ltd. (Chengdu, China). The micro total sulfhydryl (-SH) determination kit was purchased from Nanjing Jiancheng Bioengineering Institute. (Nanjing, China).

Adenosine 5′-triphosphate disodium salt hydrate (5′–ATP-Na_2_, with purity 99.0%), Adenosine 5′-diphosphate disodium salt (5′–ADP–Na_2_, with purity 90.0%), Guanosine 5′–monophosphate disodium salt hydrate (5′–GMP–Na_2_, with purity 99.0%), Cytidine 5′–monophosphate (5′–CMP, with purity 99.0%), Uridine 5′-monophosphate disodium salt (5′–UMP- Na_2_, with purity 99.0%), 5′-Inosinic acid disodium salt hydrate (5′–IMP–Na_2_, with purity 99.0%), Adenosine 5′-monophosphate disodium salt (5’–AMP–Na_2_, with purity 99.0%), Adenosine 5′-monophosphate disodium salt (5′-AMP-Na_2_, with purity 99.0%), hypoxanthine riboside (HxR, with purity 99.0%), hypoxanthine (Hx, with purity 99.0%) were purchased from Sigma company; methanol, potassium hydroxide, potassium dihydrogen phosphate, trichloroacetic acid, hydrochloric acid, and other chemical reagents were purchased from Chengdu Cologne Chemical Co., Ltd. (Chengdu, China).

### 2.2. Preparation of Marinated Pork

The ham bicipital muscle was released from both sides, surface fat was trimmed off, and the fascia was removed. The remaining part was cleaned and cut into 40 mm × 4 mm × 15 mm pieces. Five batches of muscle samples were prepared. Two groups were separated for each batch. Group 1 was control (without additive), and Group 2 was mixed with 0.5% (*w*/*w*) chili essential oil and 0.5% (*w*/*w*) pepper essential oil. The muscle sample in group 2 was tumbled and marinated at 4 °C for 6 h. Both of the muscle samples were sealed with polyethylene fresh-keeping film (Guangzhou miaojie daily necessities Co., Ltd., Guangzhou, China) and stored at 4 °C for 0, 2, 4, and 6 days. Before analysis, the muscle sample was chopped and frozen in liquid nitrogen for 10 min, crushed into muscle powder with an ultra-micro pulverizer (IKA company, Staufen, Germany).

### 2.3. Measurement of pH and Drip Loss

To analyze the storage stability of pork samples, the pH and drip loss of pork samples were determined. The pH was measured according to Wang, He, Zhang, Chen, and Li [11] with slight modification. Five grams of the crushed muscle powder was mixed with 45 mL deionized water and standing at room temperature (25 ± 2 °C) for 30 min. The mixture was centrifuged at 10,000× *g* for 5 min at 4 °C using a TGL-1650 centrifuge (Sichuan Shuke Instrument Co., Ltd., Chengdu, China). The pH of the supernatant was determined with a digital PHSJ-5 pH meter (Shanghai Yitian Scientific Instrument Co., Ltd., Shanghai, China), and triplicate determinations were performed for each sample.

Drip loss was a measure of muscle weight loss according to Meng et al. [19] with slight modification. A total of 25 g of pork was cut into pieces, and 5 g (M_1_) of them were used to determine drip loss. The pork piece was wrapped with double-layer filter paper (Fushun mingzheng filter paper factory, Anhui, China) and put into a 50 mL centrifuge tube. The pork sample was centrifuged at 4000× *g* for 10 min at 4 °C using a TGL-1650 centrifuge (Sichuan Shuke Instrument Co., Ltd., Chengdu, China). Immediately after centrifugation, the filter paper was removed for determining the mass of the samples after centrifugation (M_2_). Triplicate determinations were performed for each sample. The formula for calculating drip loss is as follow: Drip loss (%) = (M_1_ − M_2_)/M_1_ × 100%.

### 2.4. Determination of 5′-nucleotides

To evaluate the umami taste of the pork samples, the 5′-nucleotides in the control and the marinated pork were determined. The determination of 5′-nucleotides in pork was according to Poojary et al. [20] with some modification. An Agilent 1100 high performance liquid chromatograph (Agilent company, USA) equipped with Hypersil ODS2-C18 (4.6 mm × 250 mm, 5.0 μm) and a UV detector was used to determine the 5′-mononucleotides. An amount of 20 g of the crushed muscle powder was placed in a 50 mL plastic centrifuge tube, and then 5% TCA solution was added and mixed evenly. The solution was placed in a 4 °C refrigerator for 2 h before it was centrifuged at the speed of 2725× *g* for 10 min. The supernatant was transferred into a 100 mL beaker, and its pH was adjusted to 6.5 with 3 mol/L KOH solution. The solution was filtered with a qualitative filter paper (Fushun mingzheng filter paper factory, Anhui, China) into a volumetric flask and diluted to 50 mL with deionized water. The solution was filtered with a 0.45 μm organic filter membrane (Shanghai Xinya purification equipment Co., Ltd., Shanghai, China) and injected 10 μL into the Agilent 1100 high-performance liquid chromatograph (HPLC). The optimized gradient chromatographic conditions as follows: the ratio of mobile phase A (pure methanol) and B (0.05 mol/L KH_2_PO_4_ buffer) were 2% and 98%, 3% and 97%, 5% and 95%, 15% and 85% at 0 min, 5 min, 8 min, and 15 min, respectively. After 15 min, the ratio of mobile phase A and B was maintained at 15% and 85%. Column temperature: 30 °C, flow rate: 0.8 mL/min. Quantitative determination was performed by external standard method. All determinations were done in triplicates and expressed as mg of 5′-mononucleotide per 100 g of muscle (mg/100 g).

### 2.5. Determination of Free Amino Acids

To assess the contribution of Aspartic acid (Asp) and glutamic acid (Glu) on the umami taste of pork samples, the contents of free amino acids in the control and the marinated pork were determined. The determination of free amino acids in muscle was according to the method from Zhang, Ke, Bai, Chen, Guo, Mu, Li, Liao, Pan, and Zhao [16] with slight modification. Briefly, 100 mg of sample was precipitated with 50 mL 0.1 mol/L hydrochloric acid and extracted for 30 min with KQ3200DB ultrasonic oscillator (Kunshan Ultrasonic Instrument Co., Ltd., Kunshan, China). The mixture was centrifuged at 1744× *g* for 10 min. The supernatant was transferred to a 100 mL volumetric flask. The residue was repeatedly extracted again and combined with the supernatant after centrifugation. The supernatant was reacted with 5% 5-sulfosalicylic acid for 5 min, then the mixture was filtered with a qualitative filter paper (Fushun mingzheng filter paper factory, Anhui, China) to remove precipitation. The pH of the supernatant was adjusted to 2.2 with sodium hydroxide solution and passed through a 0.45 µm pore size microfiltration membrane. After precolumn derivatizing with phthalic dicarboxaldehyde, the filtrate was subjected to a reversed-phase high-performance liquid chromatography (RP-HPLC) SYKAM Amino Acid Analyzer S 433D (Sykam GmbH, Kleinostheim, Germany) equipped with PEEK column (4.6 × 150 mm, 7 µm, 10% cross-link) to determine the free amino acids. Triplicate experiments were performed, and the average values were reported.

### 2.6. Equivalent Umami Calculation

To evaluate the comprehensive umami taste contributed by umami amino acids and 5′-nucleotides, the equivalent umami calculation (EUC) was calculated according to Yamaguchi et al. [21]. The equation used is described as follows:

(1) $$\mathrm{EUC}=\sum a_{i}b_{i}+1218(\sum a_{i}b_{i})(\sum a_{j}b_{j})$$

where EUC-value represents the umami intensity in the given sample equivalent to the umami intensity given by 1 g of MSG (g MSG/100 g), a_i_ is the concentration (g/100 g) of umami amino acid aspartic acid (Asp) and glutamic acid (Glu); a_j_ is the concentration (g/100 g) of each umami 5′-mononucleotide (GMP, IMP, XMP, and AMP); b_i_ is the relative umami concentration for each umami amino acid to monosodium glutamate (MSG) (Glu =1; Asp= 0.077); b_j_ is the relative umami concentration for umami nucleotide to IMP (IMP = 1; GMP = 2.3; XMP = 0.61; AMP = 0.18); and 1218 is a synergistic constant based on the concentration (g/100 g) used.

### 2.7. Determination of Numb Taste Substances

Numb taste is the characteristic taste of pepper (*Zanthoxylum bungeanum*). It is mainly induced by the taste substances hydroxyl-α-sanshool and hydroxyl-β-sanshool. To evaluate the numb taste change of marinated pork, the content of hydroxyl-α-sanshool and hydroxyl-β-sanshool were determined according to the method of Yang et al. [22] with some modification. An amount of 10 g of the crushed muscle powder was poured into a conical bottle containing 50 mL pure methanol and extracted with a Kq3200db CNC ultrasonic cleaner (Kunshan Ultrasonic Instrument Co., Ltd., Kunshan, China) for 20 min. After centrifugation at 2725× *g* for 10 min, the supernatant was filtered with a 0.45 μm organic filter membrane (Shanghai Xinya purification equipment Co., Ltd., Shanghai, China), and then 10 μL of the filtrate was injected into the Agilent 1100 HPLC equipped with Agilent Zorbax SB-C18 column (4.6 × 150 mm, 5 μm). The column was eluted by acetonitrile and deionized water (5:5, *v*/*v*) at a flow rate of 1 mL/min at temperature 35 °C. The elution peaks of hydroxyl-α-sanshool and hydroxyl-β-sanshool were detected at 268 nm with a UV detector. The total numb taste substances were calculated by adding the value of hydroxyl-α-sanshool and hydroxyl-β-sanshool. Triplicate experiments were performed, and the average values were reported.

### 2.8. Determination of Capsaicin

Capsaicin is the main substance that induces the spicy taste in chili. To assess the change of spicy taste, the content of capsaicin was determined according to Sun [23] with some modification. An amount of 20 g of the crushed muscle powder was poured into a conical bottle containing 150 mL ethanol solution (60%) and extracted with a Kq3200db CNC ultrasonic cleaner (Kunshan Ultrasonic Instrument Co., Ltd., Kunshan, China) for 20 min. After centrifugation at 2725× *g* for 10 min, the supernatant was filtered with a 0.45 μm organic filter membrane (Shanghai Xinya purification equipment Co., Ltd., Shanghai, China), and then 10 μL of the filtrate was injected into the Agilent 1100 HPLC equipped with Agilent Zorbax SB-C18 column (4.6 × 150 mm, 5 μm). The column was eluted by methanol and deionized water (7:3, *v*/*v*) at a flow rate of 1 mL/min at temperature 30 °C. The elution peaks of capsaicin and dihydro capsaicin were detected at 280 nm with a UV detector. Triplicate experiments were performed, and the average values were reported.

### 2.9. Determination of Total Sulfhydryl (SH)

To evaluate the oxidation of pork protein, the content of total sulfhydryl was determined. Crushed muscle powder (1.00 g) was poured into 9 mL of precooled 0.86% normal saline. The mixture was embedded in an ice bath and homogenized with a FJ200-SH homogenizer (Shanghai specimen model factory, China) at 10,000 rpm for 1 min. The mixed liquid was centrifuged at 681× *g* and 4 °C for 10 min. The supernatant was mixed evenly with reagent in test kit (Nanjing Jiancheng Bioengineering Research Institute, China). After standing for 10 min, the absorbance at 412 nm was measured with a Synergy H1 microplate Reader (Biotek Instrument Co., Ltd., Winooski, VT, USA). The protein content of the supernatant was determined by the Kjeldahl method, and the content of SH was determined with a test kit (Nanjing Jiancheng Bioengineering Research Institute, Nanjing, China). The unit of SH content was mmol/gprot. Triplicate experiments were performed, and the average values were reported.

### 2.10. Determination of the Activity of Ca^2+^-ATPase

To evaluate the stability of muscle myofibrils, the activity of Ca^2+^-ATPase was determined. Crushed muscle powder (1.00 g) was mixed with 9 mL precooled 0.86% normal saline solution. The mixture was embedded in an ice bath and homogenized with an FJ200-SH homogenizer (Shanghai specimen model factory, Shanghai, China) at 10,000 rpm for 1 min. The mixed liquid was centrifuged at 681× *g* and 4 °C for 10 min. The supernatant was mixed evenly with reagent in Ca^2+^-ATPase test kit (Nanjing Jiancheng Bioengineering Research Institute, Nanjing, China) and incubated in a water bath at 37 °C for 10 min. After determining phosphorus, 0.5 mL terminator was added into the test kit to stop the reaction at room temperature. After standing for 5 min, the absorbance was measured at 636 nm with a Synergy H1 microplate Reader (Biotek Instrument Co., Ltd., Winooski, VT, USA). The unit of Ca^2+^-ATPase activity is expressed as mol Pi/mgprot/h. The activity of Ca^2+^-ATPase activity was defined as the amount of 1 μmol inorganic phosphorus produced by ATPase decomposing ATP per milligram of muscle protein per hour. Triplicate experiments were performed, and the average values were reported.

### 2.11. Soluble Peptides Analysis

To analyze the denaturation and decomposition of meat proteins, TCA-soluble peptides were determined according to Sriket et al. [24] with slight modification. Crushed muscle powder (3.00 g) was homogenized with 27 mL of 5% cold TCA solution (m/v) using a FJ200-SH homogenizer (Shanghai specimen model factory, Shanghai, China) at 10,000 rpm for 1 min. After standing in an ice bath for 60 min, the homogenate was centrifuged at 10,000× *g* for 10 min at 4 °C using a TGL-1650 centrifuge (Sichuan Shuke Instrument Co., Ltd., Chengdu, China). The supernatant was taken out to determine the TCA-soluble peptides by the Lowry method, and the result was expressed as g peptide/100 g meat.

### 2.12. Determination of Malondialdehyde (MDA)

To analyze lipid oxidation of pork samples, the MDA was determined according to the spectrophotometry method in GB5009.181-2016 [25]. Briefly, crushed muscle powder (5.00 g) was placed into a 100 mL conical flask and mixed with 50 mL TCA. The flask was sealed and shaken in an HH-6 thermostatic oscillator (Changzhou Aohua Instrument Co., Ltd., Changzhou, China) at 50 °C for 30 min. The mixture was cooled to room temperature and filtered with a qualitative filter paper (Fushun mingzheng filter paper factory, Anhui, China). The filtrate (5 mL) was mixed with 5 mL of trichloroacetic acid (TCA) in a 25 mL plugged colorimetric tube and incubated in an HH-6 thermostatic oscillator (Changzhou Aohua Instrument Co., Ltd., Changzhou, China) at 90 °C for 30 min. The mixture was cooled to room temperature and the absorbance was detected at 532 nm with a Synergy H1 microplate Reader (Biotek Instrument Co., Ltd., Winooski, VT, USA). Triplicate experiments were performed, and the average values were reported.

### 2.13. Determination of Total Volatile Base Nitrogen

To evaluate the effect of chili and pepper essential oils on the storage stability of pork, the total volatile base nitrogen (TVB-N) was determined according to the method from Sun et al. [26]. The muscle sample was prepared as follows: crushed muscle powder (5.00 g) was mixed with 75 mL of deionized water and soaked for 30 min. The TVB-N value was expressed by mg/100 g.

### 2.14. Microbiological Analysis (Total Viable Counts)

To evaluate the effect of chili and pepper essential oils on the storage stability of pork, the total viable counts were determined according to the plate colony counting method in GB4789.2-2016 [27]. Briefly, 25 g muscle samples and 225 mL sterile phosphate buffer were homogenized for 2 min using a MX-S beating homogenizer (BIOTANG Inc., Lexington, MA, USA). Then, the solutions were diluted in a 10-fold gradient, and the optimal 2 dilutions were selected for plate counting. Among them, plate counting agar (PCA, GuoYao, China) was used, and inverted culture was carried out. The incubation temperature was 37 °C for 24 h. The result was reported as lg (cfu/g).

### 2.15. Determination of Volatile Flavor Compounds

To evaluate the change of volatile flavor compounds of pork samples, the volatile flavor compounds of the crushed pork were determined according to Ting et al. [28] and Zhang et al. [29]. A 7890B-5977A gas chromatography–mass spectrometer (Agilent Technologies Inc., Santa Clara, CA, USA) was used to identify and quantify the volatile compounds in the pork. A HP-5MS UI capillary column (length 30 m, inner diameter 0.25 mm, coating 0.25 µm; Agilent, Santa Clara, CA, USA) was used as the capillary column for GC separation. Helium was used as the carrier gas at a constant flow rate of 1.0 mL/min. The temperature program employed was 2.0 min at 70 °C, a ramp of 3 °C/min to 100 °C, then raised to 130 °C at 5 °C/min and held for 3 min, then raised to 160 °C at 5 °C/min and held for 2 min, and then raised to 200 °C at 5 °C/min and held for 5 min. The mass spectrometer was operated in the electron impact mode (EI) at 70 eV (ion source temperature: 230 °C). Samples were recorded in full scan mode (m/z 35–500), the emission current was 100 μA, and the detection voltage was 350 V. The 0.01 mol/L 2,4,6-trimethylpyridine was taken as internal standard. Identification of the volatile compounds in the pork was achieved by comparing its mass spectra and retention times to the mass spectrum library NIST14.0, and then the compounds matching degree higher than 80% was reported. The absolute content of unknown compounds (*C_i_*) and its odorant activity value (*OAV*) were calculated as follow:

(2) $$C_{i}=\frac{\rho \times V\times A_{i}}{A\times m}\;$$

(3) $$OAV=\frac{C_{i}}{OT_{i}}$$

where *C_i_* is the absolute content of unknown compounds, μg/kg; *ρ* is the mass concentration of internal standard (2 μg/μL); *V* is the volume of internal standard; *A_i_* is the peak area of each component; *A* is the peak area of internal standard substance; *m* is the mass of sample (kg); *OT_i_* is the threshold of the compound.

### 2.16. Statistical Analysis

All the tests were conducted in triplicates. The results were analyzed by ANOVA at a significance level of 5% (H0: *p* < 0.05). The comparison of means was analyzed by Fisher’s LSD tests using the SAS statistical package.

## 3. Results and Discussion

### 3.1. Effect of Conditioning on the Storage Stability of Pork

#### 3.1.1. pH and Drip Loss

**Figure 1:**
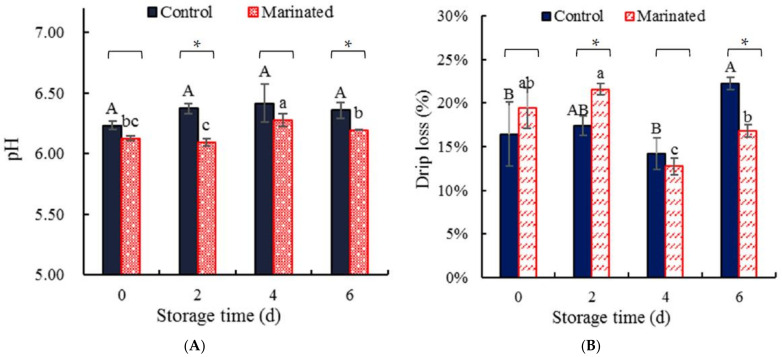
The pH (**A**) and drip loss (**B**) of the marinated pork and the control. Values are presented as mean ± standard deviation. Different letters above the bars highlighting the standard deviations indicate significant differences (*p* < 0.05). The asterisk indicates significant differences (*p* < 0.05) between treatments for each time.

The pH in Figure 1A shows that the pH values of the marinated pork and the control were all increased with the storage time increase, but the pH values of the control did not significantly (*p* > 0.05) differ from each other; the pH of the marinated pork stored at the fourth day was significantly (*p* < 0.05) higher than others. The pH values of the control were higher than those of the marinated pork samples, and the pH values of the control stored at the second day and fourth day were significantly (*p* < 0.05) higher than those of the marinated pork. The pH values of the marinated pork and the control were increased with the storage time increase. This was consistent with that of Meng, Sun, Shi, Cheng, and Fan [19]. They attributed the increase of pH to the degradation of meat protein into volatile alkaline nitrogen molecules (e.g., amines). The results indicated that the protein degradation of the control was more than those of the marinated pork.

Figure 1B shows drip losses of the control were not significantly (*p* > 0.05) changed within four days, but increased significantly (*p* < 0.05) at the sixth day. The drip loss of the marinated pork was not significantly (*p* > 0.05) changed within two days, but decreased significantly (*p* < 0.05) at the fourth day and then increased significantly (*p* < 0.05) at the sixth day. The drip loss of the control was significantly (*p* < 0.05) lower than that of the marinated pork at the second day, but the drip loss of the control was significantly (*p* < 0.05) higher than that of the marinated pork. The drip loss is an index to show muscle to hold inherent water, which demonstrates destabilization and disruption of muscle cell membranes, myofibrillar shrinkage, net charge effect, etc. [30]. The marinate processing of the marinated pork might break the native structure of the pork muscle, and this may lead to the drip loss of the marinated pork being higher than those of the control at 0 and 2 d. The protein degradation of the control was higher than that of the marinated pork, which might be the reason that the drip loss of the control was higher than that of the marinated pork at 4 and 6 d. To further confirm the protein degradation of the control and the marinated pork, the content of sulfhydryl, the soluble peptides, and the activity of Ca^2+^-ATPase were determined.

#### 3.1.2. Content of Sulfhydryl (SH)

**Figure 2:**
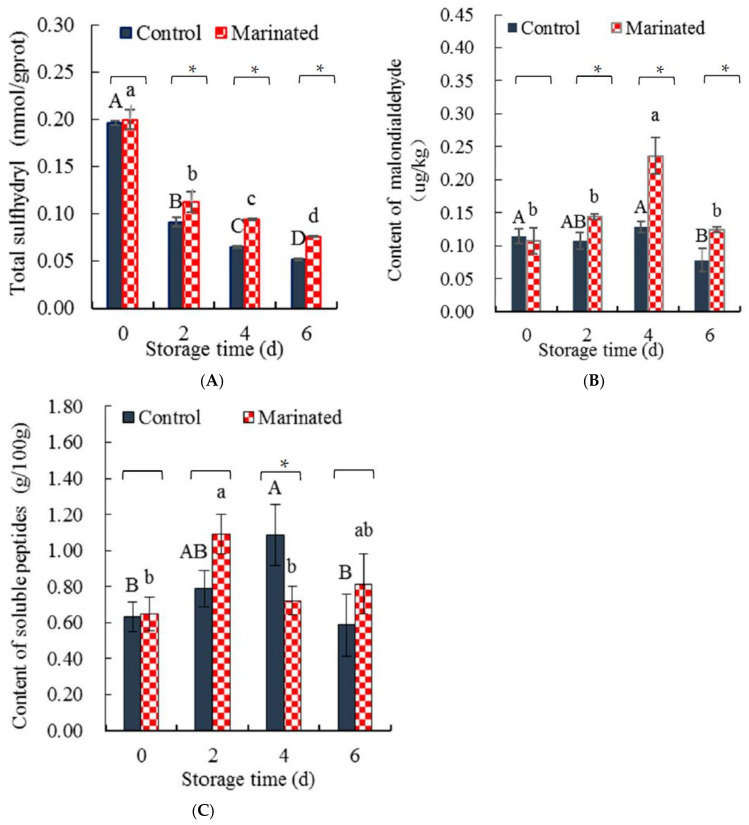
The content of sulfhydryl groups (**A**), MDA (**B**), and soluble peptide (**C**) in the marinated pork and the control. Values are presented as mean ± standard deviation. Different letters above the bars highlighting the standard deviations indicate significant differences (*p* < 0.05). The asterisk indicates significant differences (*p* < 0.05) between treatments for each time.

Thiol or SH groups are reactive groups in meat proteins that can be oxidized to form disulfide bonds, resulting in the loss of SH [31]. Accordingly, the content of SH groups is frequently used to indicate the denaturation of meat protein caused by processing, thermal, or storage treatment [32]. Figure 2A shows that the content of SH in the marinated pork and control samples decreased significantly (*p* < 0.05) as storage time increased, whereas SH content in the marinated pork did not significantly (*p* > 0.05) differ from that of the control at 0 d. However, the content of SH in the marinated pork was significantly (*p* < 0.05) higher than that of the control at 2, 4, and 6 days. The rate of the SH decrease for the marinated pork (0.021 mmol/(gprot·d)) was lower than that of the control (0.024 mmol/(gprot·d)).

Compared with the control, the marinated pork was supplemented with 0.5% (*w*/*w*) chili essential oil and 0.5% (*w*/*w*) pepper essential oils. Both phenolic compounds and unsaturated bonds are present in the compounds, which can inhibit the oxidation of sulfhydryl and cause the chili or pepper essential oil to exhibit antioxidant effects [33,34]. The oxygen free radical scavenging efficiency of 0.1% pepper essential oil was similar to that of 0.1% vitamin C [35]. The scavenging effect of the chili or pepper essential oil might be the reason for the higher content of SH in the marinated pork than in the control. The content of sulfhydryl in the control were lower than that of the marinated pork at 2, 4, and 6 days further confirmed that the protein degradation of the control was more than those of the marinated pork.

#### 3.1.3. Content of MDA

MDA is a by-product of lipid oxidation and is used to indicate the deterioration of meat quality [36]. The decrease in SH content (Figure 2A) in the marinated and control groups suggested the occurrence of protein oxidation. To further confirm the oxidation and deterioration of pork muscle, the MDA content in the marinated meat and control meat was determined (Figure 2B). As shown in Figure 2B, the MDA content in the marinated pork and the control increased and then decreased with increasing storage time. However, the MDA content in the marinated pork was higher than that in the control and reached its highest level on the fourth day of refrigeration. A similar increase was observed in beef stored at refrigeration temperature [37].

Chili and pepper essential oils were added in marinated pork, and these essential oils consist of plant extracts and oil. The oil in the chili and pepper essential oils was oxidized, ultimately generating MDA [38], which may justify the higher MDA content in the marinated pork than the control after the second day (Figure 2B). MDA is a byproduct of lipid oxidation and can further react with myofibril proteins [36], which might justify the lower MDA content of marinated pork and the control on the sixth day relative to the fourth day. This speculation is supported by a significantly lower content of SH on day 6 than on day 4 (Figure 2A).

#### 3.1.4. Soluble Peptides

The soluble peptide content indicates the denaturation and decomposition of meat proteins [39,40]. The contents of soluble peptides in the marinated pork and control are shown in Figure 2C. Based on Figure 2C, the content of soluble peptide increased with increasing storage time; however, this content was the highest on the second day for the marinated pork and the fourth day for the control. The soluble peptide content of the marinated pork showed similar trends with that of the drip loss (Figure 1B); the soluble peptide content of the control showed reverse trends with that of drip loss (Figure 1B) at the fourth and sixth days.

The marinated pork was tumbled and marinated during preparation. Such treatment destroyed the original muscle tissue and induced oxidization. The dual action of muscle tissue destruction and oxidation might result in the soluble peptides of the marinated pork being one day earlier, up to the highest level, compared to that of the control. The drip loss of chilled pork increased with increasing storage time [41]. Herein, some soluble peptides were lost from the drip, which might explain the lower content of soluble peptide in the marinated pork on the fourth and sixth days relative to that on the second day, and the lower content of soluble peptide in the control on the sixth day relative to that on the fourth day.

#### 3.1.5. Activity of Ca^2+^-ATPase

**Figure 3:**
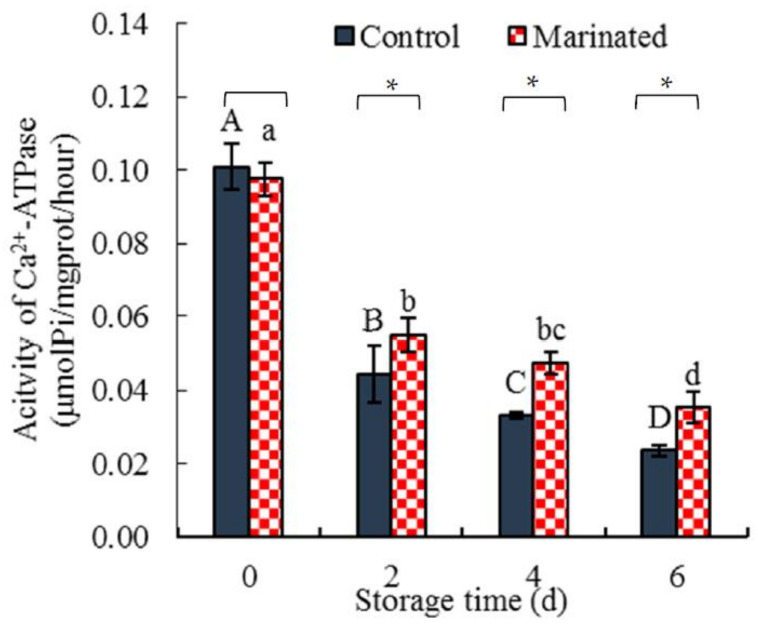
The activity of Ca^2+^-ATPase in the marinated pork and control. Values are presented as mean ± standard deviation. Different letters above the bars highlighting the standard deviations indicate significant differences (*p* < 0.05). The asterisk indicates significant differences (*p* < 0.05) between treatments for each time.

The activity of Ca^2+^-ATPase is used to indicate the stability of muscle myofibrils during processing or storage [42,43]. Figure 3 shows that the activity of Ca^2+^-ATPase in the marinated pork and control samples decreased significantly (*p* < 0.05) with increasing storage time; however, the activity of Ca^2+^-ATPase in the marinated pork did not significantly (*p* > 0.05) differ from that of the control at 0 d. Nonetheless, at 2, 4, and 6 days, the activities of Ca^2+^-ATPase in the marinated pork were significantly (*p* < 0.05) higher than that in the control. The decrease rate of Ca^2+^-ATPase activity of the marinated pork (0.010 U/(mg prot ·d)) was lower than that of the control (0.013 U/(mg prot ·d)).

Muscle myofibril Ca^2+^-ATPase is localized in the heads of myosin and is required for the interaction between myosin and actin [44]. The lower activity of Ca^2+^-ATPase indicates denaturation of the muscle myofibrils [43]. As a result, the decrease in the activity of Ca^2+^-ATPase in the marinated pork was lower than that in the control, suggesting that the muscle myofibrils of the marinated pork were less denatured. This speculation is supported by the linear correlation (r = 0.999) between the activity of Ca^2+^-ATPase and SH content. The results of the content of sulfhydryl and the muscle myofibril Ca^2+^-ATPase in the control and the marinated pork further confirmed that the protein degradation of the control was more than those of the marinated pork.

#### 3.1.6. Total Volatile Base Nitrogen and Viable Counts

**Figure 4:**
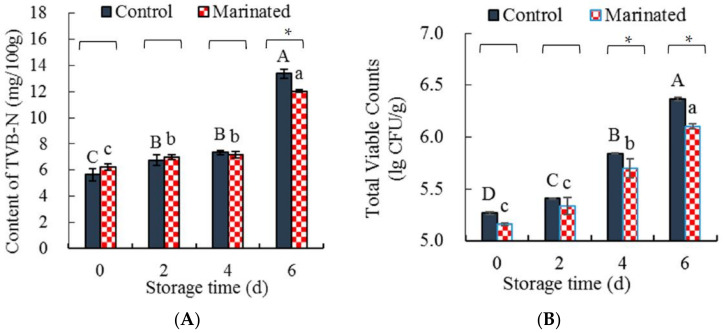
The TVB-N (**A**) and viable count (**B**) of the marinated pork and the control. Values are presented as mean ± standard deviation. Different letters above the bars highlighting the standard deviations indicate significant differences (*p* < 0.05). The asterisk indicates significant differences (*p* < 0.05) between treatments for each time.

To evaluate the effect of chili and pepper essential oils on the storage stability of pork, the TVB-N content and viable count of the marinated pork and control were determined (Figure 4). As depicted in Figure 4A, the TVB-N of the marinated pork and the control increased significantly (*p* < 0.05) as the storage time increased and were the highest on day 6. A similar increasing trend was observed in pork treated with *P. oleracea* extract [45]. TVB-N is an index used to evaluate the deterioration of meat products [46]. When the TVB-N content is higher than 15 mg/100 g, meat or meat products are not safe to consume [47]. For this study, the TVB-N contents of the marinated pork and the control were 12.04 mg/100 g and 13.37 mg/100 g, respectively. These results indicated that the marinated pork and the control were safe to consume when refrigerated for six days.

Figure 4B shows that the total microorganisms in the marinated pork (R^2^ = 0.9981) and the control (R^2^ = 0.9972) were quadratically correlated with the increase in storage time, and the total number of microorganisms in the control increased significantly (*p* < 0.05) from 0 to 6 d. Similar results were reported for black pepper essential oil-treated fresh pork [48]. The total microorganism content of the marinated pork was lower than that of the control from 0 to 6 days and increased significantly (*p* < 0.05) from 2 to 6 days. These results suggest that the chili and pepper essential oils were effective at inhibiting the growth of microorganisms in marinated pork. It has been reported that the chili and pepper essential oils were effective at inhibiting the growth of spoilage bacteria [48,49], which might explain the lower total microorganisms in the marinated pork being than in the control from 0 to 6 d.

According to [47], the pork is not safe to be consumed when it has a total microorganism count greater than 6 lg (CFU/g). The total microorganisms in the marinated pork and the control were 6.1 lg (CFU/g) and 6.4 lg (CFU/g), respectively (Figure 4B). As both the marinated and control meat had values greater than 6 lg (CFU/g) after 6 days of storage, they were not suitable for consumption. The quadratic correlated equations of the marinated pork (y = 0.0144x^2^ + 0.0728x + 5.1539) and the control (y = 0.0242x^2^ + 0.0408x + 5.2588) were used to predict the time when the total microorganisms exceeded 6.0 lg(CFU/g); 4.76 d and 5.54 d were found for the control and the marinated pork, respectively. The refrigeration time of the marinated pork was extended by 16.58% by treatment with the chili and pepper essential oils. Therefore, chili and pepper essential oils are effective at inhibiting the growth of microorganisms during short-term refrigeration (not more than 5.54 d).

### 3.2. Non-Volatile Taste

Umami, numb, and spicy tastes are three characteristic and favored tastes in China; the spices that induce numb and spicy tastes have antibacterial function and are viewed as natural antibiotics [48,49]. Based on these features, they are frequently added to meat products to enhance the flavor [1,12]. Therefore, the key flavor substances were determined to evaluate the taste changes in the marinated pork during storage.

#### 3.2.1. Umami Taste

The umami taste is a very important flavor for meat and other food products [17,29,50]. The umami taste not only improves food flavor, but also improves the nutrition intake of elderly people and patients, protecting against duodenal cancer, reducing ingestion of sodium chloride, and decreasing consumption of fat [51,52]. Umami amino acids and nucleotides are the main umami ingredients in meat products; the umami amino acids are Glu and Asp, and the umami nucleotides are IMP, GMP, CMP, UMP, and AMP [52]. To evaluate the umami taste, umami amino acids and nucleotides were determined (Table 1). Based on Table 1, the GMP, IMP, XMP, and EUC values of the control and marinated pork decreased significantly (*p* < 0.05) as the storage time increased; however, the GMP, IMP, and XMP contents of the marinated pork were higher than those of the control on the second, fourth, and sixth days. The EUC value of the marinated pork did not significantly (*p* > 0.05) change before the second day and remained higher than that of the control from days 2 to 6. The AMP content of the control and marinated pork decreased significantly (*p* < 0.05) on the second day, increased significantly (*p* < 0.05) on the fourth day, and decreased significantly (*p* < 0.05) on the fifth day. The AMP content in the control was lower than that in marinated pork. The Glu content in the marinated pork increased significantly (*p* < 0.05) as the storage time increased but fluctuated from days 2 to 6 in the control. The content of Asp in the marinated pork increased significantly (*p* < 0.05) on the second day and then decreased significantly (*p* < 0.05) from days 4 to day 6. There was no significant change (*p* > 0.05) on the second day; however, the Asp content increased significantly (*p* < 0.05) on the fourth day and decreased significantly (*p* < 0.05) on the sixth day. By using the EUC value as an index to indicate the umami taste change, the umami taste of the control and marinated pork was found to decrease with increasing storage time; however, the decreased rate of the marinated pork (0.080) was lower than that of the control (0.0783). The ECU value of the marinated pork was significantly (*p* < 0.05) higher than that of the control at second, 4th and 6th d. Therefore, chili and pepper essential oils were conducive to inhibiting the decrease in the umami taste of pork.

After slaughter, the carcasses of pigs undergo four processes: carcass stiffness, aging, autolysis, and deterioration, which are complicated biochemical reactions [53]. In theory, the decomposition and production of nucleotides in meat are highly related to the metabolism of muscle cells [54]. GMP is mainly produced by XMP under the catalysis of GMP synthase (EC: 6.3.5.2), IMP is mainly produced by AMP under the catalysis of AMP deaminase (EC: 3.5.4.6), and IMP is decomposed to XMP and Hx [54,55]. Therefore, the changes in the nucleotides in the control and marinated pork might be related to the metabolism of muscle cells and the activities of endogenous enzymes. However, these factors require further analysis.

#### 3.2.2. Numb Taste

**Figure 5:**
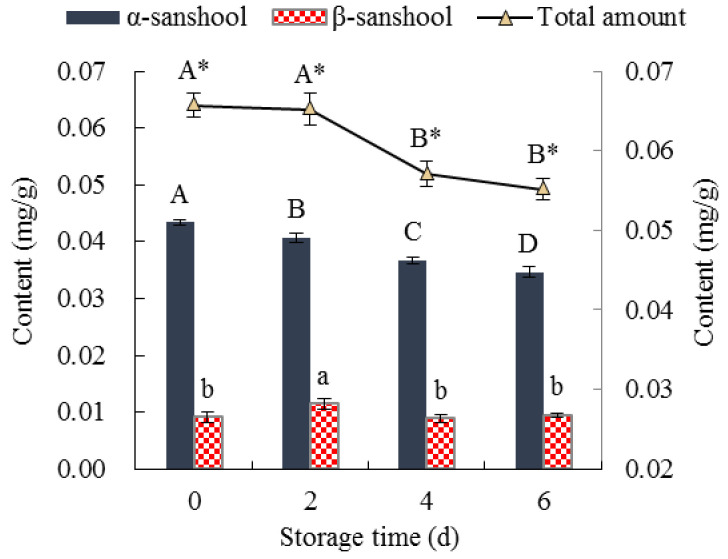
Numb taste of the marinated pork during cold storage. Values are presented as mean ± standard deviation. Different letters above the bars or points highlighting the standard deviation indicate significant differences (*p* < 0.05). The asterisk indicates differences between capital letters.

Numb taste is a typical flavor of Sichuan cuisine-marinated meat products owing to the addition of pepper or its essential oil [56]. Hydroxyl-α-sanshool and hydroxyl-β-sanshool are the key flavor substances in pepper [57]. Therefore, to analyze the changes in the numb taste of the marinated pork during cold storage, the contents of hydroxyl-α-sanshool and hydroxyl-β-sanshool were determined (Figure 5). As shown in Figure 5, the hydroxyl-α-sanshool content decreased significantly (*p* < 0.05) as the storage time increased. In fact, the hydroxyl-β-sanshool content increased significantly (*p* < 0.05) on the second day but decreased significantly (*p* < 0.05) from the second day to the sixth day. The total contents of hydroxyl-α-sanshool and hydroxyl-β-sanshool decreased significantly (*p* < 0.05) from the second day to the sixth day.

There are four unsaturated bonds in hydroxyl-α-sanshool and hydroxyl-β-sanshool, which are easily oxidized when exposed to air for a long time [58]. The oxidation of unsaturated bonds might cause the decrease in the total content of hydroxyl-α-sanshool and hydroxyl-β-sanshool with increasing storage time. Such a finding was consistent with and further supported the higher SH content of the marinated pork than the control (Figure 2A). The hydroxyl-α-sanshool could be converted to hydroxyl-β-sanshool in the oxidation procedure [59,60]. The conversion of hydroxyl-α-sanshool to hydroxyl-β-sanshool and continuous oxidation might be the reason for the increase in the content of hydroxyl-β-sanshool on the second day, which then decreased significantly with increasing storage time.

#### 3.2.3. Spicy Taste

**Figure 6:**
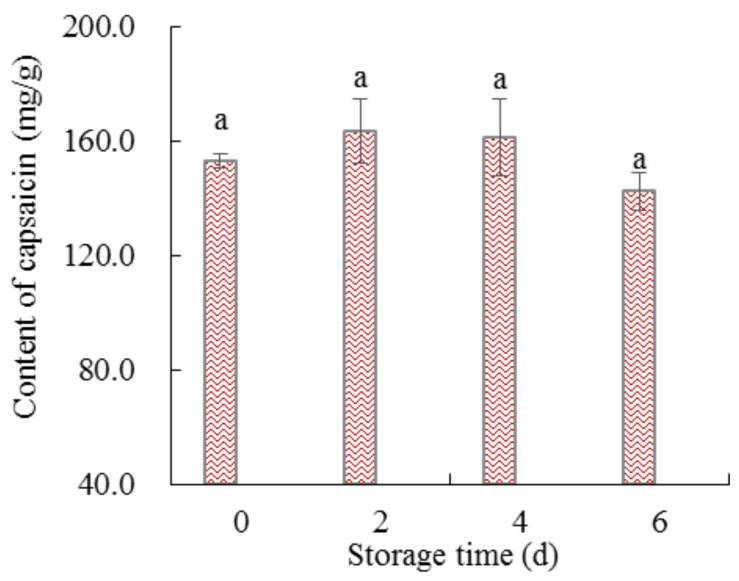
The content of capsaicin in the marinated pork. Values are presented as mean ± standard deviation. Different letters above the bars highlighting the standard deviations indicate significant differences (*p* < 0.05).

Spicy taste is another commonly used flavor in the conditioning of meat products. The spicy taste is caused by the addition of chili powder or its essential oil [61]. Capsaicin is the main substance that stimulates the tongue buds to produce a spicy taste. Therefore, to analyze the change in spicy taste of the marinated pork during cold storage, the content of capsaicin in the marinated pork was determined (Figure 6). As shown in Figure 6, the content of capsaicin did not significantly (*p*> 0.05) change with the increase in refrigeration time, but its content decreased on the sixth day compared to other time points.

The decomposition of capsaicin is highly related to environmental conditions; less air exposure and lower humidity and temperature could inhibit its decomposition [62]. The chili essential oil was tumbled and used to marinate pork, which was then packed. Capsaicin infiltrated pork and reduced contact with air. As the storage time increased, more air had contact with the pork, resulting in partial capsaicin decomposition; this might be the reason for the non-significant change in capsaicin content (*p* > 0.05) with the increase in refrigeration time, but the content decreased on the sixth day.

### 3.3. Volatile Flavor Compounds

Volatile flavor compounds are easily smelted as they are unpacked. Accordingly, these compounds are highly related to the sensory quality of foods [63]. To evaluate the volatile flavor of the marinated pork, the flavor compounds were determined (Table 2). Table 2 shows that six alcohol compounds (3-Methyl-1-butanol, (2R, 3R)-(-)-2,3-Butanediol, 1-Nonanol, 1-Octen-3-ol, l-alanine, 2-(aziridin-1-yl) ethanamine, 5-Methyl-2-hexanone), four ketone compounds (5-Methyl-2-hexanone, 4-Isopropyl-1-methyl-1-cyclohexen-3-one, octane, and tetradecane), and one ester compound (isoamyl acetate) were present. The categories of volatile compounds increased with increasing storage time up to the maximum, as the pork was stored for six days.

The oxidation of lipids and proteins, catabolism of amino acids, and microbial activity are the three main factors that influence volatile flavor compounds in food [64,65]. The SH content of the marinated pork was the lowest on day six, and the TVB-N and viable count of the marinated pork were the highest on day six. These results may explain why the volatile flavor category in marinated pork was the highest on day six. As the volatile flavor compounds of the marinated pork were found to be the highest on day six, this finding further revealed the deterioration of the marinated pork during refrigeration and storage for six days.

## 4. Conclusions

The content of SH and the activity of Ca^2+^-ATPase of the marinated pork were significantly (*p* < 0.05) higher than that of the control on the second, fourth, and sixth days. This demonstrated that the addition of chili essential oil and pepper essential oil inhibited the oxidation and denaturation of pork protein. The TVB-N and viable count of the marinated pork were significantly (*p* < 0.05) lower than that of the control on the sixth day. These results confirmed the antimicrobial effect of the chili essential oil and pepper essential oil. The non-volatile flavors (umami, numb, and spicy) of the marinated pork decreased with increasing refrigeration time; the decomposition of umami-enhancing nucleotides (GMP, IMP, and XMP), and the number of flavor substances (hydroxyl-α-sanshool and hydroxyl-β-sanshool) and spicy (capsaicin) taste compounds caused the decrease in non-volatile flavors. The volatile compounds in the marinated pork were the highest after the six days of storage.

## Figures and Tables

**Table 1:** The umami amino acids and the nucleotides in the marinated pork and the control (mg/100 g).

Meat Category	Umami Ingredients	0 d	2 d	4 d	6 d
**Control**	5′-GMP	2.28 ± 0.00 A	1.11 ± 0.00 B	0.39 ± 0.00 C	0.34 ± 0.04 C
	5′-IMP	137.31 ± 0.00 A	54.85 ± 1.03 B	17.87 ± 0.00 C	6.01 ± 0.00 D
	5′-AMP	5.88 ± 0.00 A	4.27 ± 0.03 D	5.66 ± 0.00 B	5.31 ± 0.00 C
	5′-XMP	15.38 ± 0.01 A	7.21 ± 0.08 B	3.57 ± 0.00 C	1.78 ± 0.00 D
	Asp	3.65 ± 0.15 B	3.70 ± 0.10 B	4.92 ± 0.07 A	3.29 ± 0.07 C
	Glu	2.70 ± 0.00 C	4.65 ± 0.15 B	1.93 ± 0.04 D	7.14 ± 0.06 A
	EUC	0.56 ± 0.00 A	0.38 ± 0.02 B	0.06 ± 0.00 C	0.08 ± 0.00 C
**Marinated pork**	5′-GMP	2.25 ± 0.00 a	1.47 ± 0.01 b *	0.70 ± 0.01 c *	0.55 ± 0.01 d *
	5′-IMP	135.15 ± 0.05 a *	84.15 ± 0.19 b *	41.2 ± 0.01 c *	10.74 ± 0.44 d *
	5′-AMP	5.95 ± 0.00 a	5.21 ± 0.06c *	5.56 ± 0.01 b	5.17 ± 0.07 d *
	5′-XMP	14.53 ± 0.01 a *	8.04 ± 0.00b *	4.34 ± 0.01 c *	3.60 ± 0.15 d *
	Asp	3.74 ± 0.02 c	5.6 ± 0.00a *	4.6 ± 0.10 b *	4.32 ± 0.12 b *
	Glu	2.91 ± 0.13 c	4.75 ± 0.05 b	4.95 ± 0.05 b *	5.76 ± 0.01 a *
	EUC	0.58 ± 0.02 a	0.59 ± 0.01 a *	0.30 ± 0.00 b *	0.11 ± 0.00 c *

Means in the same row with different letters differ significantly (*p* < 0.05). The asterisk indicates significant differences (*p* < 0.05) between treatments for each time.

**Table 2:** Changes in the volatile flavor compounds in the marinated pork.

No.	RT (min)	CAS	Name	Formula	Content (μg/kg)				Threshold Value (μg/kg)	OVA			
					0 d	2 d	4 d	6 d		0 d	2 d	4 d	6 d
**1**	3.31	123-51-3	3-Methyl-1-butanol	C_5_H_12_O	/	140.07 ± 3.53	/	575.29 ± 25.26	250	/	0.56	/	2.30
**2**	4.32	24347-58-8	(2R,3R)-(-)-2,3-Butanediol	C_4_H_10_O_2_	/	/	/	25.28 ± 1.11	95.10	/	/	/	0.27
**3**	4.48	111-65-9	Octane	C_8_H_18_	/	/	/	43.31 ± 1.90	10000	/	/	/	0.00
**4**	6.7	123-92-2	Isoamyl acetate	C_7_H_14_O_2_	/	/	/	10.10 ± 0.44	2	/	/	/	5.05
**5**	6.8	56-41-7	L-Alanine	C_3_H_7_NO_2_	5.34 ± 0.07	/	/	/	2.6	2.05	/	/	/
**6**	7.01	143-08-8	1-Nonanol	C_9_H_20_O	/	/	/	8.77 ± 0.39	/	/	/	/	/
**7**	7.09	110-12-3	5-Methyl-2-hexanone	C_7_H_14_O	/	/	/	49.33 ± 2.17	330	/	/	/	0.15
**8**	8.04	4025-37-0	2-(aziridin-1-yl)ethanamine	C_4_H_10_N_2_	/	/	/	6.61 ± 0.29	/	/	/	/	/
**9**	10.1	3391-86-4	1-Octen-3-ol	C_8_H_16_O	5.28 ± 0.07	9.99 ± 0.25	2.33 ± 0.04	5.47 ± 0.24	1.5	3.52	6.66	1.55	3.65
**10**	19.87	89-81-6	4-Isopropyl-1-methyl -1-cyclohexen-3-one	C_10_H_16_O	3.20 ± 0.04	2.73 ± 0.07	/	/	680	0.00	0.00	/	/
**11**	25.02	629-59-4	Tetradecane	C_14_H_30_	27.56 ± 0.38	57.93 ± 1.46	20.54 ± 0.34	16.84 ± 0.74	1000	0.03	0.06	0.02	0.02

RT—retention time; OVA—odorant activity value.

## Data Availability

Data are contained within the article.
